# Cross-linking, Immunoprecipitation and Proteomic Analysis to Identify Interacting Proteins in Cultured Cells

**DOI:** 10.21769/BioProtoc.3258

**Published:** 2019-06-05

**Authors:** Hao Wang, Meiling He, Belinda Willard, Qingyu Wu

**Affiliations:** 1 Department of Cardiology, Shanghai Institute of Cardiovascular Diseases, Zhongshan Hospital, Fudan University, Shanghai, China; 2 Department of Cardiovascular & Metabolic Sciences, Lerner Research Institute, Cleveland Clinic, Cleveland, USA; 3 Cyrus Tang Hematology Center, Collaborative Innovation Center of Hematology, State Key Laboratory of Radiation Medicine and Prevention, Soochow University, Suzhou, China; 4 Proteomics Core, Lerner Research Institute, Cleveland Clinic, Cleveland, USA

**Keywords:** Corin, Immunoprecipitation, N-glycosylation, Protein cross-linking, Protein-protein interaction, Proteomic analysis, Serine protease

## Abstract

Extracellular expression is essential for the function of secreted and cell surface proteins. Proper intracellular trafficking depends on protein interactions in multiple subcellular compartments. Co-immunoprecipitation and the yeast two-hybrid system are commonly used to investigate protein-protein interactions. These methods, however, depend on high-affinity protein interactions. In many glycoproteins, glycans are important for protein intracellular trafficking and extracellular expression. If glycoprotein interactions are transient and relatively weak, it may be challenging to use co-immunoprecipitation or the two-hybrid system to identify glycoprotein-binding partners. To circumvent this problem, protein cross-linking can be applied first to immobilize the transient and/or low-affinity protein interactions. Here we describe a protocol of protein cross-linking, co-immunoprecipitation, and proteomic analysis, which was used to identify endoplasmic reticulum (ER) chaperones critical for the folding and ER exiting of N-glycosylated serine proteases in human embryonic kidney (HEK) 293 cells. This approach can be used to identify other protein interactions in a variety of cells.

## Background

Natriuretic peptides play a key role in body fluid homeostasis (Kuhn, 2016; Matsuo *et al.*, 2019). Corin is a transmembrane protease expressed on the surface of cardiac myocytes, where it activates the natriuretic peptides to regulate blood pressure and cardiac function (Yan *et al.*, 2000; Zhou and Wu, 2014). Corin is synthesized as a zymogen (Knappe *et al.*, 2003), which is converted to an active enzyme by proprotein convertase subtilisin/kexin-6 (PCSK6) on the cell surface (Chen *et al.*, 2015; Chen *et al.*, 2018). Naturally occurring mutations that impair corin cell surface expression and activation have been found in patients with hypertension and heart disease (Wang *et al.*, 2012; Dong *et al.*, 2013 and 2014; Zhang *et al.*, 2014 and 2017).

Human corin is a glycoprotein with 19 N-glycosylation sites (Yan *et al.*, 1999; Hooper *et al.*, 2000). N-glycosylation is essential for the cell surface expression and activation of corin (Liao *et al.*, 2007; Gladysheva *et al.*, 2008). In site-directed mutagenesis studies, a corin mutant without N-glycans in the protease domain had reduced levels on the cell surface due to impaired intracellular trafficking (Wang *et al.*, 2015 and 2018). Further immune staining experiments showed that the mutant protein was retained in the ER (Wang *et al.*, 2018). These results suggest that N-glycans on corin, particularly those in the protease domain, may interact with other ER proteins that are critical for corin intracellular trafficking (Wang *et al.*, 2018). Identification of such ER proteins should help to understand the cellular mechanism in regulating corin expression and function.

Co-immunoprecipitation and the yeast two-hybrid system are commonly used to analyze protein-protein binding and complex formation (Berggard *et al.*, 2007; Kaboord and Perr, 2008). These methods are suitable mostly for studying stable and/or high-affinity protein-protein interactions. In many cases, however, protein interactions in specific subcellular compartments are transient and unstable. In glycoprotein synthesis, for example, transient N-glycan-protein interactions are essential for glycoprotein folding and subsequent ER exiting (Ellgaard and Frickel, 2003; Lamriben *et al.*, 2016). The traditional methods such as protein co-immunoprecipitation and the two-hybrid system may not be suitable for studying such N-glycan-protein interactions. To circumvent this problem, protein cross-linking can be applied to immobilize the transient and/or weak protein interactions before co-immunoprecipitation proceeds.

In a recent study, we designed a protocol of protein cross-linking, co-immunoprecipitation, and proteomic analysis to examine the role of N-glycans in corin intracellular trafficking. We expressed corin wild-type (WT) and a mutant lacking the N-glycosylation site in the protease domain (N1022Q) in separate HEK293 cells. The cells were treated with dithiobis succinimidyl propionate (DSP), a cell membrane permeable cross-linker, which has an amine-reactive *N*-hydroxysuccinimide (NHS) ester at each end of a cleavable spacer (Mattson *et al.*, 1993). NHS esters react with primary amines in the side chain of lysine residues and the N-termini of proteins, thereby forming stable amide bonds connecting co-localized proteins (Mattson *et al.*, 1993). Proteins in HEK293 cells cross-linked to corin WT and the mutant were isolated by immunoprecipitation. After breaking the disulfide bond in the spacer of DSP under reducing conditions, proteins were separated by SDS-PAGE and analyzed by in-gel digestion and liquid chromatography-mass spectrum (LC-MS). By comparing proteins that were differentially bound to corin WT and the mutant, we identified calnexin as a key ER chaperone that mediates the N-glycan-dependent folding and ER exiting of corin and other N-glycosylated serine proteases such as enteropeptidase and prothrombin (Wang *et al.*, 2018).

## Materials and Reagents

1. Cell lifters (Corning, catalog number: 3008)
2. Surgical blades (any brand)
3. Pipette tips (any brand)
4. 1.5 ml microcentrifuge tubes (any brand)
5. 150 mm cell culture dishes (Corning, catalog number: 430599)
6. HEK293 cells (ATCC, catalog number: CRL-1573)
7. pcDNA 3.1/V5-His-based plasmids (Thermo Fisher, catalog number: K480001)
8. Dulbecco’s modified Eagle’s medium (DMEM) (Lerner Research Institute Cell Culture Core, catalog number: 11-500)
9. Fetal bovine serum (FBS) (Corning, catalog number: 35-011-CV)
10. Phosphate buffered saline (PBS), 10x (Affymetrix, catalog number: 75889)
11. Dithiobis succinimidyl propionate (DSP) (Thermo Fisher, catalog number: 22585)
12. Dimethyl sulfoxide (DMSO) (Thermo Fisher, catalog number: 24600)
13. Glycine (Research Products International (RPI), catalog number: G36050)
14. Tris-base (Fisher Scientific, catalog number: 502-13-709)
15. Sodium chloride (RPI, catalog number: S23020)
16. Nonidet P-40 (Affymetrix, catalog number: 19628)
17. Protease inhibitor cocktail (Sigma-Aldrich, catalog number: P8340)
18. Protein assay dye reagent (Bio-Rad, catalog number: 500-0006)
19. Bovine serum albumin (BSA) (Sigma-Aldrich, catalog number: A9647)
20. Anti-V5 antibody (Thermo Fisher, catalog number: R96025)
21. Protein A-Sepharose (Thermo Fisher, catalog number: 10-1042)
22. Sodium dodecyl sulfate (SDS) (Fisher Scientific, catalog number: BP166500)
23. SDS-PAGE protein sample buffer (2x) (Bio-Rad, catalog number: 1610737)
24. β-mercaptoethanol (Fisher Scientific, catalog number: BP176-100)
25. Pre-stained protein ladder (Thermo Fisher, catalog number: 26616)
26. Tris-Glycine gel (4-20%) (Thermo Fisher, catalog number: XP04200BOX)
27. Silver staining kit (Thermo Fisher, catalog number: 24600)
28. Ethanol (any brand, HPLC grade)
29. Acetic acid (any brand, HPLC grade)
30. Acetonitrile (any brand, HPLC grade)
31. SilverQuest silver staining kit (Thermo Fisher, catalog number: LC607)
32. Dithiothreitol (DTT) (Thermo Fisher, catalog number: R0861)
33. Iodoacetamide (Thermo Fisher, catalog number: A39271)
34. Trypsin, sequencing grade (Thermo Fisher, catalog number: 90057)
35. Ammonium bicarbonate (Fisher Scientific, catalog number: A643-500)
36. Formic acid (Thermo Fisher, catalog number: 28905)
37. Fugene reagents (Promega, catalog number: E2311)
38. G418 (Teknova, catalog number: G5001)
39. DSP stock solution (see Recipes)
40. Cell lysis buffer (see Recipes)
41. SDS-PAGE buffer (see Recipes)

## Equipment

1. Pipetman P20 (Gilson, catalog number: F144801)
2. Pipetman P200 (Gilson, catalog number: F144801)
3. Pipetman P1000 (Gilson, catalog number: F144801)
4. Humidified cell culture incubator (any brand)
5. Microcentrifuge (Thermo Fisher, catalog number: 75002446)
6. Benchtop rocker (any brand)
7. Mini gel apparatus (Thermo Fisher, catalog number: A25977)
8. Spectrophotometer or microplate reader (any brand with 595 nm wavelength)
9. Power supply (Bio-Rad, catalog number: 1645052)
10. Speedvac (Savant, AES1010-120)
11. Ultimate Nano-3000 HPLC (Dionex, RSLCnano 3000)
12. LTQ-Obitrap hybrid mass spectrometer system (Thermo Scientific; Elite)
13. Trapping column: Dionex 2 cm x 75 μm id Acclaim Pepmap C18, 5 μm, 100 Å reversed-phase capillary (Thermo Fisher, catalog number: 164564)
14. Analytical column: Dionex 15 cm x 75 μm id Acclaim Pepmap C18, 2 μm, 100 Å reversed-phase capillary chromatography column (Thermo Fisher, catalog number: 164534)
15. Ice bucket (any brand)

## Software

1. Xcaliber version 2.2 (Thermo Scientific)
2. UniProtKB (https://www.uniprot.org/help/uniprotkb), downloaded on June 29, 2016
3. Mascot version 2.3.0 (Matrix Science)
4. Proteome Discoverer 2.2 (Thermo Scientific)
5. Scaffold version 4.0.6.1 (Proteome Software)

## Procedure

*Notes:*

1. *
pcDNA 3.1/V5-His-based plasmids expressing human corin WT and the N1022Q mutant were described previously (Wang et al., 2015). The corin proteins encoded by these plasmids contain a C-terminal V5 tag that is recognized by an anti-V5 antibody.
*
2. *
The corin-expressing plasmids were transfected into HEK293 cells (authenticated by STR DNA profiling, no mycoplasma contamination) using Fugene reagents. The cells were cultured in DMEM with 10% FBS and 400 μg/ml of G418 at 37 °C in a humidified incubator with 5% CO_2_ to select stable corin-expressing cell clones. The experimental procedures were described previously (Wang et al., 2018).
*

- Protein cross-linking in cells
  1. Seed HEK293 cells stably expressing corin WT and the mutant N1022Q in two separate 150 mm dishes with DMEM and 10% FBS (~5 x 10^6^ cells/dish in 25 ml medium).
*
Note: In the mutant N1022Q, the N-glycosylation site at N1022 in the protease domain was mutated (Figure 1).
*
    **Figure 1.**
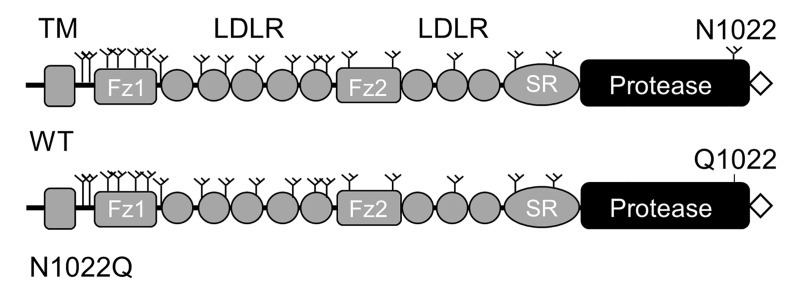
Domain structures of corin WT and the mutant N1022Q. Corin consists of an N-terminal cytoplasmic tail, a transmembrane domain (TM) and an extracellular region containing two frizzled (Fz) domains, eight LDL receptor (LDLR) repeats, a scavenger receptor (SR) domain, and a serine protease domain. Y-shaped symbols indicate N-glycosylation sites. In the mutant N1022Q, the N-glycosylation site at N1022 in the protease domain is mutated. Recombinant corin proteins contain a C-terminal V5 tag (diamond shape) used for immunoprecipitation.
  2. Culture the cells at 37 °C in a humidified incubator with 5% CO_2_ until the cells reach ~90% of confluency (~36 h).
  3. Remove the medium by suction and wash the cells once with PBS (pre-chilled on ice, 10 ml per dish).
*
Note: Figure 2 illustrates the protocol flow chart in the experiments of protein cross-linking, immunoprecipitation, SDS-PAGE, in-gel digestion, and LC-MS proteomic analysis, which are described below.
*
    **Figure 2.**
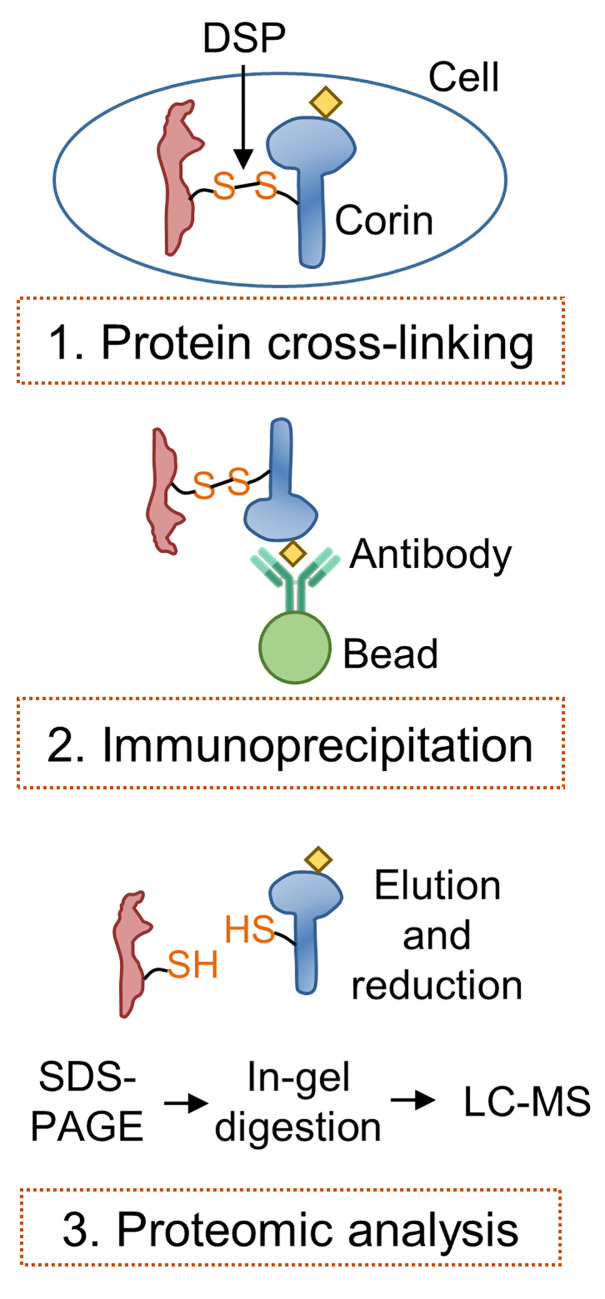
Protocol flow chart. In protein cross-linking, recombinant corin protein containing a C-terminal V5 tag (diamond shape) in HEK293 cells was cross-linked with interacting proteins (brown), using dithiobis succinimidyl propionate (DSP). In immunoprecipitation, corin-associated proteins were isolated using an anti-V5 antibody and protein A-Sepharose beads. In proteomic analysis, corin-associated proteins were eluted from the beads under reducing conditions and analyzed by SDS-PAGE followed by in-gel trypsin digestion and LC-MS analysis. The experiments were done in parallel in HEK293 cells expressing corin WT and the mutant N1022Q. Proteins that were differentially associated with corin WT and the mutant N1022Q were selected for further biochemical and cellular studies.
  4. Add DSP (0.8 mg/ml in PBS, 10 ml per dish) to the cells and incubate at 4 °C for 30 min.
*
Note: The chemical structure of DSP is shown in Figure 3.
*
    **Figure 3.**
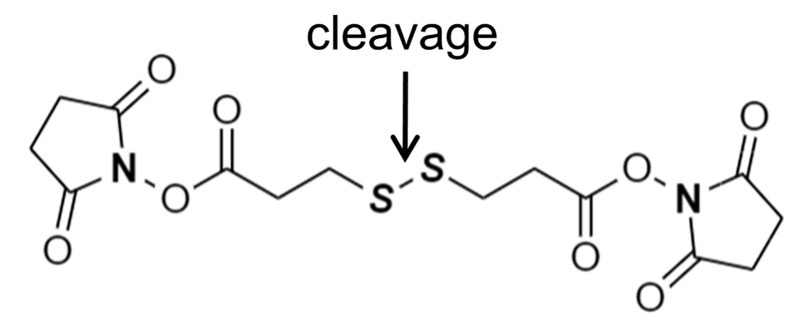
Chemical structure of DSP. The chemical structure of the homobifunctional cross-linker DSP is shown. The indicated disulfide bond will be broken upon reduction.
  5. Stop the cross-linking reaction by adding 1 ml of PBS containing 0.2 M glycine at 4 °C and incubate for 15 min. No need to remove the DSP solution.
  6. Discard the reaction solution and gently wash the cells with 10 ml pre-chilled PBS.
  7. Use a cell lifter to scrap the cells off the dish in 1.5 ml PBS and transfer the cells in suspension to a 1.5 ml microcentrifuge tube.
  8. Spin down the cells with a microcentrifuge (900 *× g* at 4 °C for 5 min) and discard the supernatant.
- Immunoprecipitation of bait (corin)-associated proteins
  1. Lyse the pelleted cells in a cell lysis buffer containing Nonidet P-40 (see Recipes) (750 μl) at 4 °C for 30 min.
*Note: Tap the tube every 5 min to ensure that the cells are completely lysed.*
  2. Centrifuge the cell lysate at 16,200 *× g*, 4 °C, for 10 min.
  3. Collect the supernatant (cell lysate) and discard the insoluble cellular debris.
  4. Take 1 μl of the cell lysate sample to measure the protein concentration using the protein assay dye kit and a spectrophotometer.
  5. Transfer the cell lysate (2,000 μg of total proteins) to a new microcentrifuge tube and dilute the sample to 1 ml in the cell lysis buffer.
  6. Immunoprecipitate corin proteins (bait) and associated proteins (prey) in the cell lysate by incubating with an anti-V5 antibody (1:1000) and protein A-Sepharose beads (50 μl) at 4 °C for 2 h on a benchtop rocker (with gentle shaking).
  7. Spin down the beads at 16,200 *× g*, 4 °C, for 1 min and remove the supernatant.
  8. Wash the beads twice with pre-chilled PBS (1 ml, 5 min each).
  9. Remove PBS by centrifugation (16,200 *× g*, 4 °C, 1 min).
  10. Elute proteins from the beads and dissociate the bait-prey protein complexes with SDS-PAGE sample buffer (2x, 50 μl) containing 5% β-mercaptoethanol at 37 °C for 30 min.
  11. Spin down the beads at 16,200 *× g*, 4 °C, for 1 min and collect the supernatant.
- Analysis of the eluted proteins by SDS-PAGE
  1. Load the entire eluted protein sample (50 μl) onto a 4-20% gradient Tris-Glycine gel, together with PageRuler^TM^ Prestained Protein Ladder (5 μl).
  2. Run the electrophoresis in SDS-PAGE buffer at a voltage ≤ 160 V.
  3. Stop the electrophoresis before the dye front runs out of the gel.
  4. Visualize proteins on SDS-PAGE gels using a commercial silver staining kit (Thermo Fisher), following the manufacturer’s instructions (Figure 4).
    **Figure 4.**
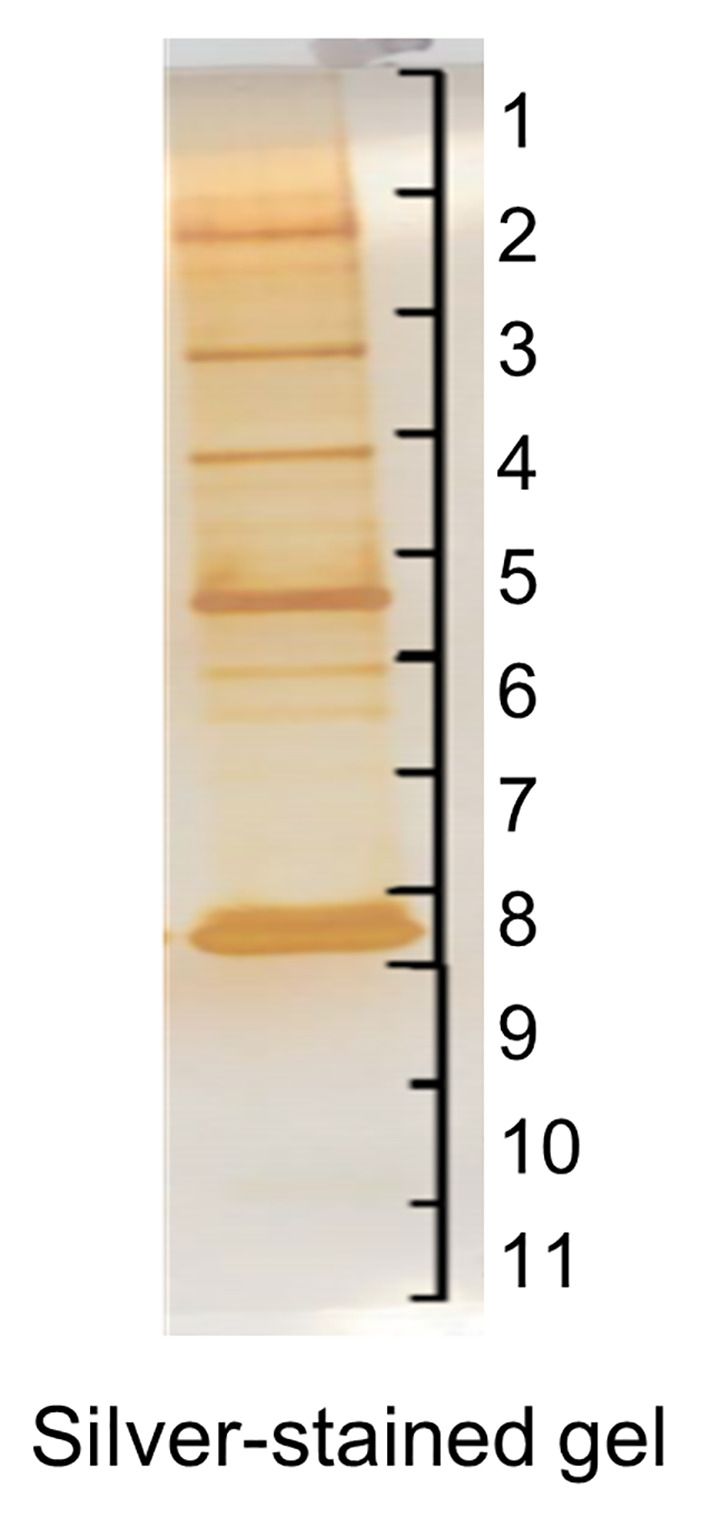
An example of silver-stained gel for proteomic analysis. Proteins eluted from Sepharose beads were analyzed by SDS-PAGE under reducing conditions followed by silver staining to visualize protein bands. The gel was cut horizontally into 11 slices, which were subjected to further in-gel trypsin digestion and LC-MS analysis.
- In-gel digestion of proteins
  1. Cut the stained gel into slices using a surgical blade (Figure 4).
  2. Transfer each gel slice into a microcentrifuge tube. These gel pieces are then cut into 1 mm^3^ pieces.
  3. Wash gel pieces with 200 μl deionized water and de-stain with SilverQuest silver staining kit according to manufacturer’s instructions. Wash away the destain reaction solution with 5 x 175 μl deionized water.
  4. Dehydrate gel pieces with 175 μl acetonitrile for 5 min. Remove acetonitrile and dry the gel pieces in Speedvac for 3 min. Reduce proteins with 50 μl DTT (5 mg/ml in 100 mM ammonium bicarbonate) for 30 min at room temperature. Remove DTT. Alkylate proteins with 50 μl iodoacetamide (25 mg/ml in 100 mM ammonium bicarbonate) for 30 min at room temperature. Remove iodoacetamide.
  5. Dehydrate gel pieces by adding 175 μl acetonitrile and incubate for 5 min. Remove acetonitrile. Rehydrate gel pieces in 175 μl 100 mM ammonium bicarbarbonate. Remove ammonium bicarbonate. Dehydrate gel pieces with 175 μl acetonitrile for 5 min. Remove acetonitrile and dry gel pieces in Speedvac for 3 min.
  6. Add 15 μl of trypsin (10 ng/μl in 50 mM ammonium bicarbonate) to the tube and incubate at room temperature overnight to ensure complete protein digestion. After overnight digestion, add 30 μl extraction buffer (50% acetonitrile/5% formic acid). Mix and incubate for 10 min. Transfer the supernatant to an Eppendorf tube. Add a second 40 μl aliquot of extraction buffer, mix and combine supernatants.
  7. Reduce the volume of extract to < 10 μl in Speedvac.
  8. Resuspend the extract in 1% acetic acid to a final volume of ~30 μl for LC-MS analysis.
- LC-MS proteomic analysis
  1. Inject 5 μl of the peptide extract into the LC-MS system and load onto the trapping column for 5 min at a flow rate of 10 μl/min.
  2. Elute the peptides from the trapping and reversed-phase columns at a flow rate of 0.3 μl/min with a binary gradient starting at 95% A (0.1% formic acid) and 5% B (acetonitrile/0.1% formic acid) for 5 min followed by a linear increase from 2-40% B in 85 min (Figure 5). The column is washed by ramping to 80% B and holding for 5 min prior to re-equilibration of the column at 2% B for 15 min.
    **Figure 5.**
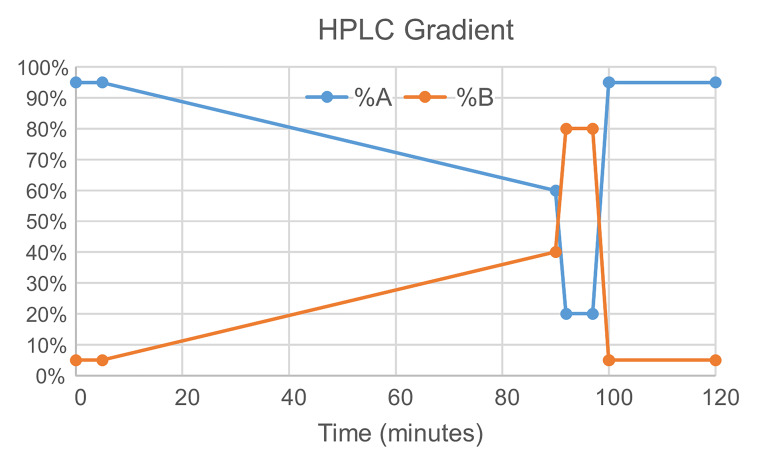
Illustration of a binary gradient in HPLC analysis
*
Note: An example of peptide elution profile is shown in Figure 6.
*
    **Figure 6.**
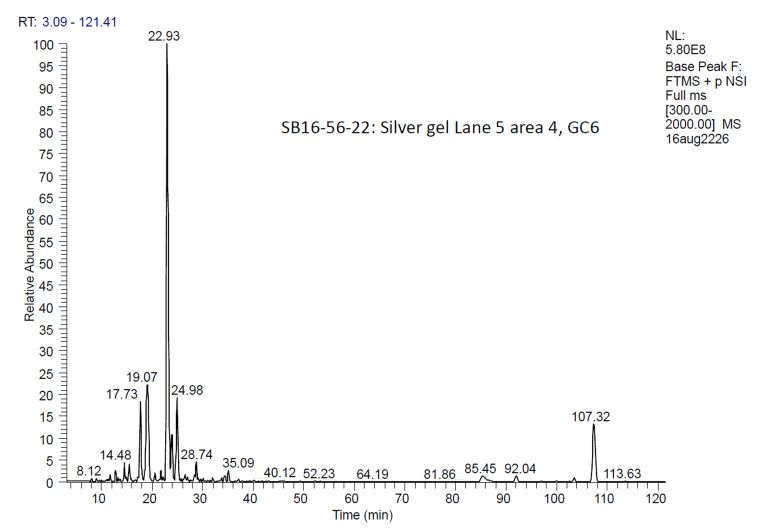
A protein elution profile from one of the tryptic digests. Most of the tryptic peptides were eluted during the linear gradient from 2 to 40% acetonitrile.
  3. The source conditions include a spray voltage of 2.0 kV and a capillary temperature of 220 °C. Other source parameters including tube lens, S-lens, and multipole voltages should be optimized regularly.
  4. The Orbitrap Elite instrument is operated using the XCaliber Software package. Analyze the tryptic digests using a data-dependent acquisition method with the following parameters. Acquire full MS1 scans at a resolution of 60,000 in the Orbitrap MS. Select peptides for MS/MS analysis using a Top15 method with dynamic exclusion enabled (3 repeats in 50 s, exclusion duration of 90 s). Acquire MS/MS spectra in the ion trap with an isolation width of 3.0 Da and a collision energy of 35%.
  5. Search the data using the programs Mascot and Sequest-HT which is bundled into Proteome Discoverer 1.4. To use Mascot, convert the RAW data files to mascot generic files (mgf files) using Proteome Discoverer 1.4. Search the data against the human UniProtKB database with both Mascot and Sequest using the following parameters: MS mass tolerance of 10 ppm, MS/MS mass tolerance of 0.6 Da, trypsin as the protease with full specificity, 2 missed cleavage sites, oxidized methionine as a variable modification, and carbamidomethylation as a fixed modification. Perform additional searches using the program Sequest specifically against the sequence of the bait protein to identify post-translational modifications such as phosphorylation, acetylation, and ubiquitination.
  6. Perform protein and peptide validation by uploading the Proteome Discoverer (msf files) and Mascot (dat files) search results into the program Scaffold. Utilize Scaffold to perform additional database searches using X!Tandem with the same parameters as described above. Perform False Discover Rate analysis by searching the data against a reversed UniProtKB database and filter identifications based on a peptide level FDR of 0.1% and protein level FDR of 1%. Only consider proteins identified by two peptides, one of which is unique.

## Data analysis

The spectral counts in the LC-MS proteomic analysis (Step E) were examined. Proteins with high spectral counts were possible bait-interacting candidates. All proteins with spectral counts ≥ 10 were included for further analysis. To identify the proteins that differentially interacted with corin WT and the mutant, a ratio of ≥ 2-fold difference in spectral counts between WT and the mutant was used as a selection criterion. The selected candidates were analyzed for their subcellular expression patterns by searching the human UniProtKB database (www.uniprot.org/uniprot), which includes information on protein subcellular locations. The proteins that are predominantly expressed in the targeted subcellular location, *i.e.*, ER in our study, were selected and tested in additional biochemical and cellular experiments (Wang *et al.*, 2018).

*Note: The criterion for protein spectral counts and the ratio between the control and the targeted protein may be modified depending on experimental settings.*

## Notes

1. The protein expression system (in Step A1), including the plasmids and the cells, may be modified depending on the target, *i.e.*, bait, protein(s) of interest. The antibody used in immunoprecipitation (in Step B6) should also be modified based on the specific experimental design.
2. The LC-MS proteomic analysis was done at the Proteomic Core of the Lerner Research Institute of the Cleveland Clinic.

## Recipes

1. DSP stock solution
  13 mM DSP in dimethyl sulfoxide
  Store at 4 °C for ≤1 week
2. Cell lysis buffer
  50 mM Tris-base (pH 8.0)
  150 mM sodium chloride
  1% Nonidet P-40 (vol/vol)
  1% protease inhibitor cocktail (vol/vol)
3. SDS-PAGE buffer
  25 mM Tris-base
  250 mM glycine
  3.5 mM SDS
